# A Rare Case of Multimetastatic Choriocarcinoma

**DOI:** 10.3390/diagnostics16162539

**Published:** 2026-08-12

**Authors:** Daria Szymaniuk, Paula Szymaniuk, Maciej Janica, Emilia Świenc, Paweł Muszyński

**Affiliations:** 1 Department of Radiology, Śniadeckiego Voivodeship Hospital in Bialystok, Curie 26, 15-950 Bialystok, Poland; 2 University Clinical Hospital in Białystok, Curie 24a, 15-276 Bialystok, Poland; 3 Department of Cardiology, Lipidology and Internal Diseases, Medical University of Bialystok, Żurawia 14, 15-540 Bialystok, Poland

**Keywords:** choriocarcinoma, metastatic choriocarcinoma, ultra high risk choriocarcinoma

## Abstract

Choriocarcinoma is a highly malignant trophoblastic tumour characterised by the secretion of human chorionic gonadotropin (β-hCG). Depending on its origin, choriocarcinoma is divided into gestational (GCC) or nongestational (NGCC). The prognosis is poor due to its high malignancy and possible hemorrhagic metastasis to distant organs. In the present case, a 40-year-old woman presented to the ophthalmology clinic with intermittent visual disturbances and a headache. An MRI of the head revealed a small, round area of contrast enhancement, suggesting a vascular malformation. A month later, the patient presented to the emergency department after syncope. A head CT scan revealed two large intracerebral hematomas, which were evacuated by craniotomy. However, there were recurrences of intracranial bleeding, some of which were reoperated. During hospitalisation, chest X-ray and CT scans revealed a mass in the right middle lobe of the lung. Successive subcapsular hematomas were detected in both liver lobes; the patient underwent two laparotomies. A gynaecological examination and transvaginal ultrasonography excluded the possibility of pregnancy despite the elevated levels of βhCG. Histopathological examination of the liver tissue confirmed the presence of metastatic foci of choriocarcinoma. The patient received cycles of chemotherapy. After chemotherapy, βhCG decreased.

**Figure 1:**
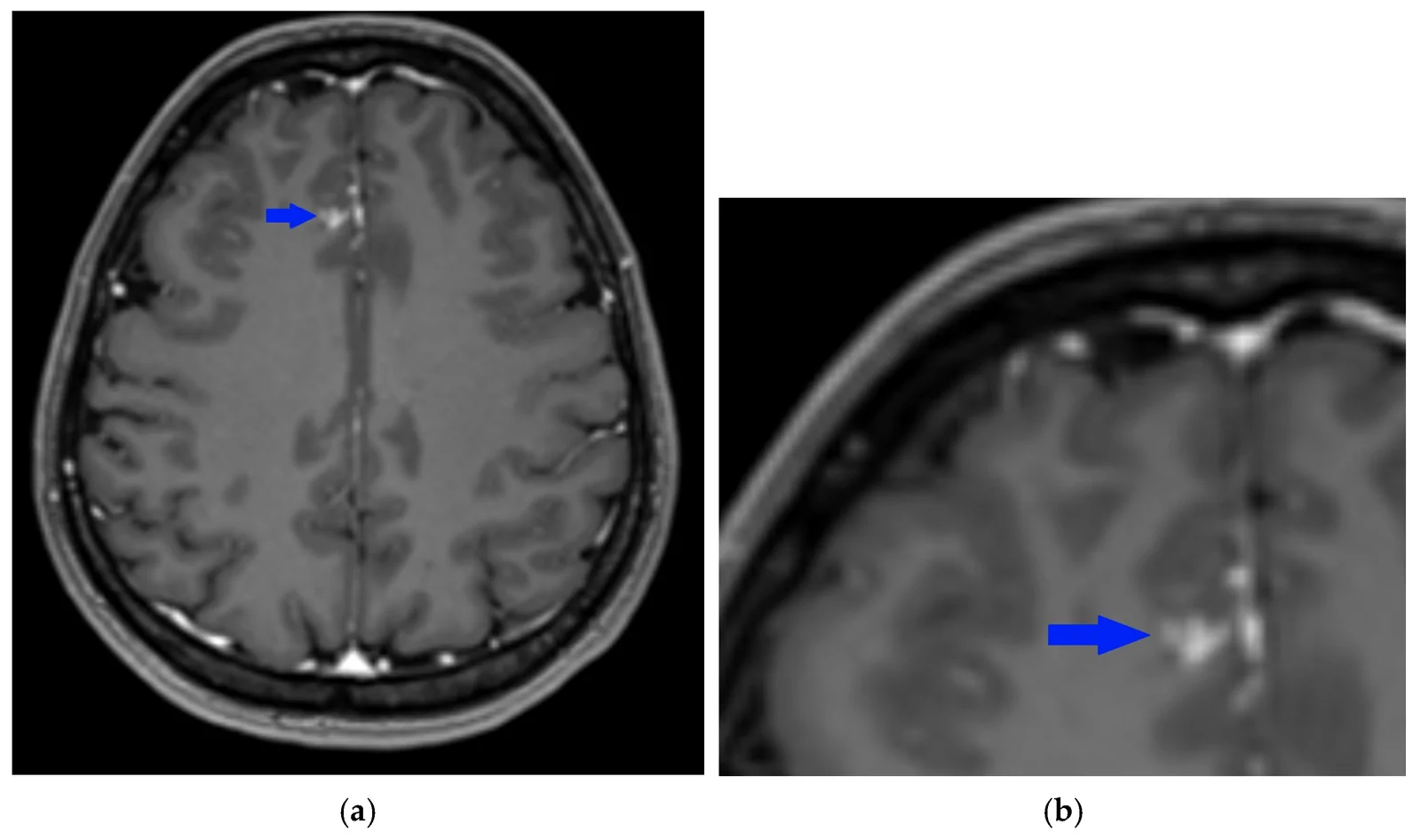
A 40-year-old woman was seen in the ophthalmology clinic complaining of intermittent visual disturbances and a headache. Brain MRI revealed a small, round area of contrast enhancement suggesting a vascular malformation—marked with blue arrow (**a**,**b**). At that time, she had one episode of intermenstrual bleeding. Apart from that, the patient had regular 28-day menstrual cycles. She had a history of one uncomplicated pregnancy and one full-term vaginal delivery five years ago.

**Figure 2:**
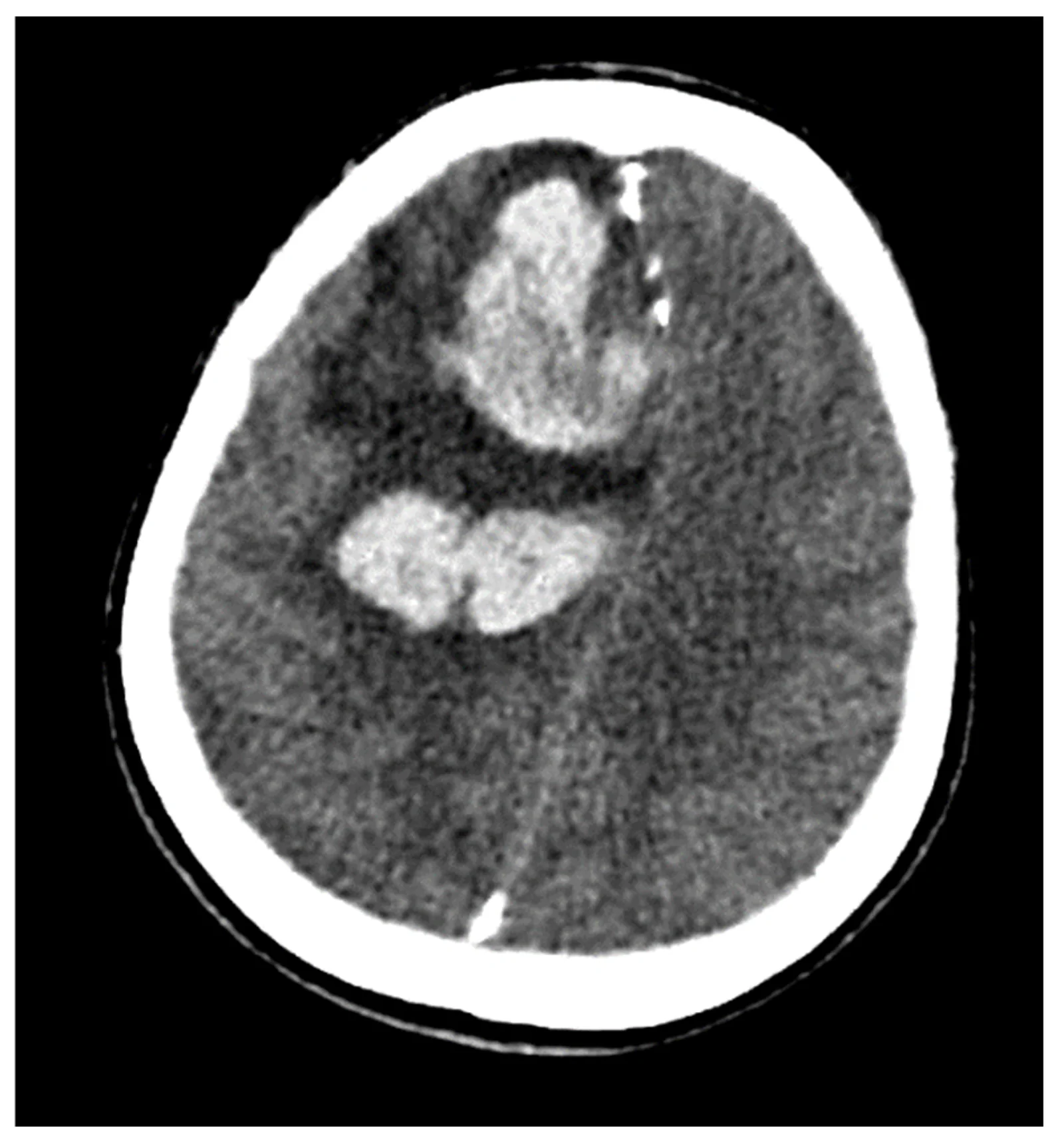
A month later, the patient presented to the emergency department by ambulance in a serious condition after losing consciousness, accompanied by nausea and vomiting. Neurological examination revealed a dilated right pupil, no reaction to light, muscle tension in the upper extremities and absent lower limb reflexes. The patient was intubated, and laboratory tests were performed. The results were negative for psychoactive substances and alcohol; other blood parameters were normal. A head CT scan revealed extensive intracranial bleeding with two large intracerebral hematomas: one in the right frontal lobe measuring 53 × 43 mm and another in the right frontoparietal region measuring 47 × 35 mm. The hematomas caused compression of the right lateral ventricle and a 15 mm midline shift to the left. A craniotomy was performed to evacuate both hematomas. The intraoperative histopathology revealed a vascular malformation. However, there were recurrent intracranial bleedings, some of which required reoperation.

**Figure 3:**
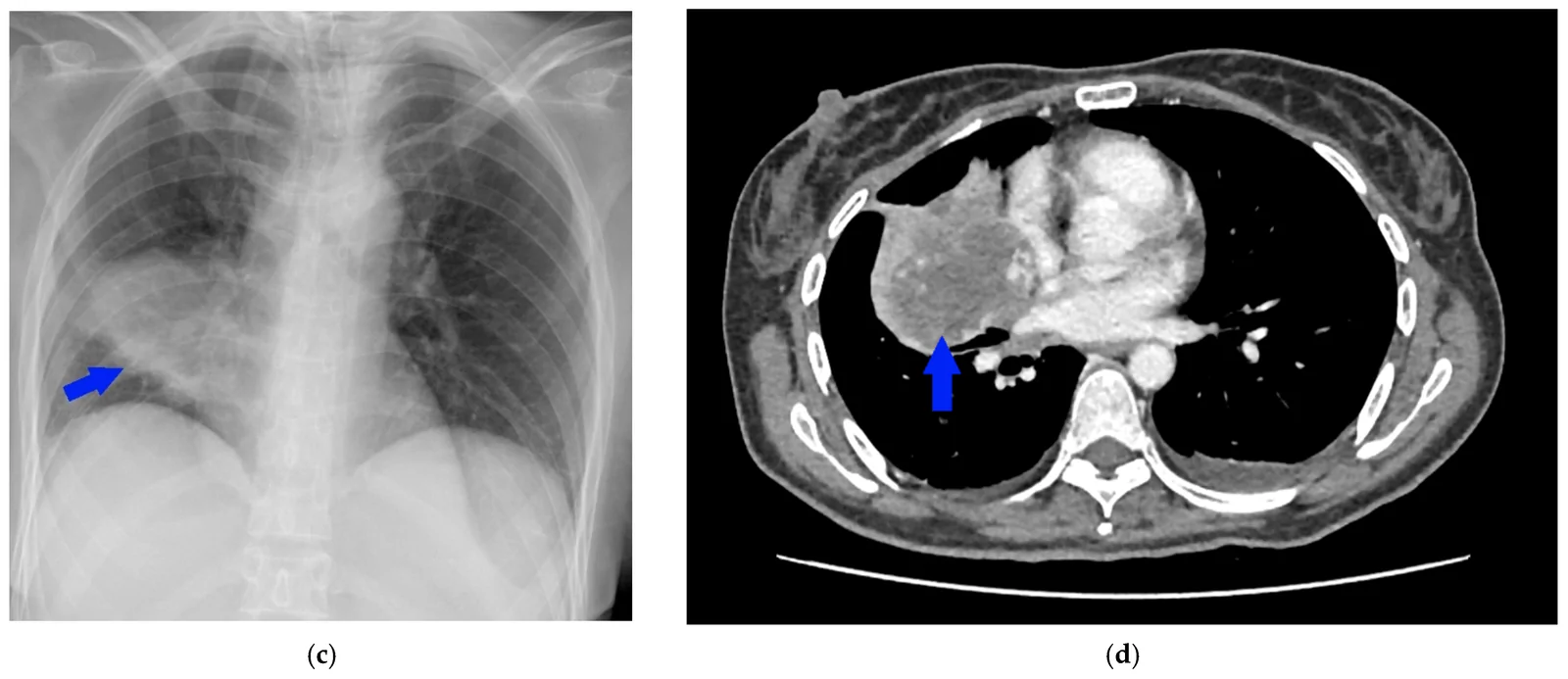
During hospitalisation, chest X-ray (**c**) and CT (**d**) scans revealed a 58 × 70 × 65 mm mass in the right middle lobe of the lung—marked with blue arrow. Due to deterioration of the patient’s condition and technical difficulties, a biopsy of the lung lesion was not performed.

**Figure 4:**
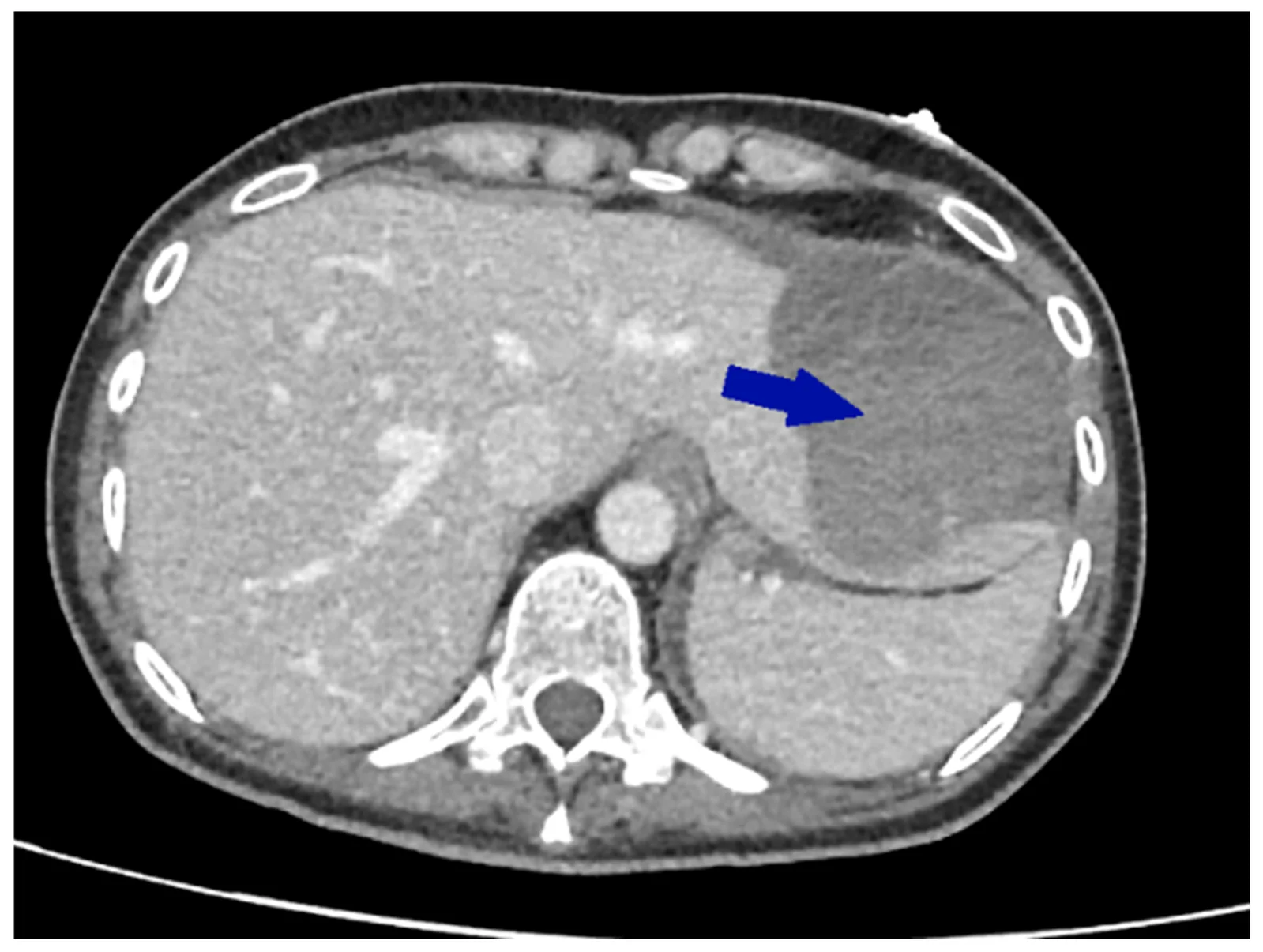
A patient reported abdominal pain while on the ward. The CT scan of the abdomen demonstrated a subcapsular haematoma measuring 99 × 77 × 126 mm in the left hepatic lobe with heterogeneous density, suggesting active haemorrhage—marked with blue arrow. Surgery was performed, and the liver haematoma was evacuated.

**Figure 5:**
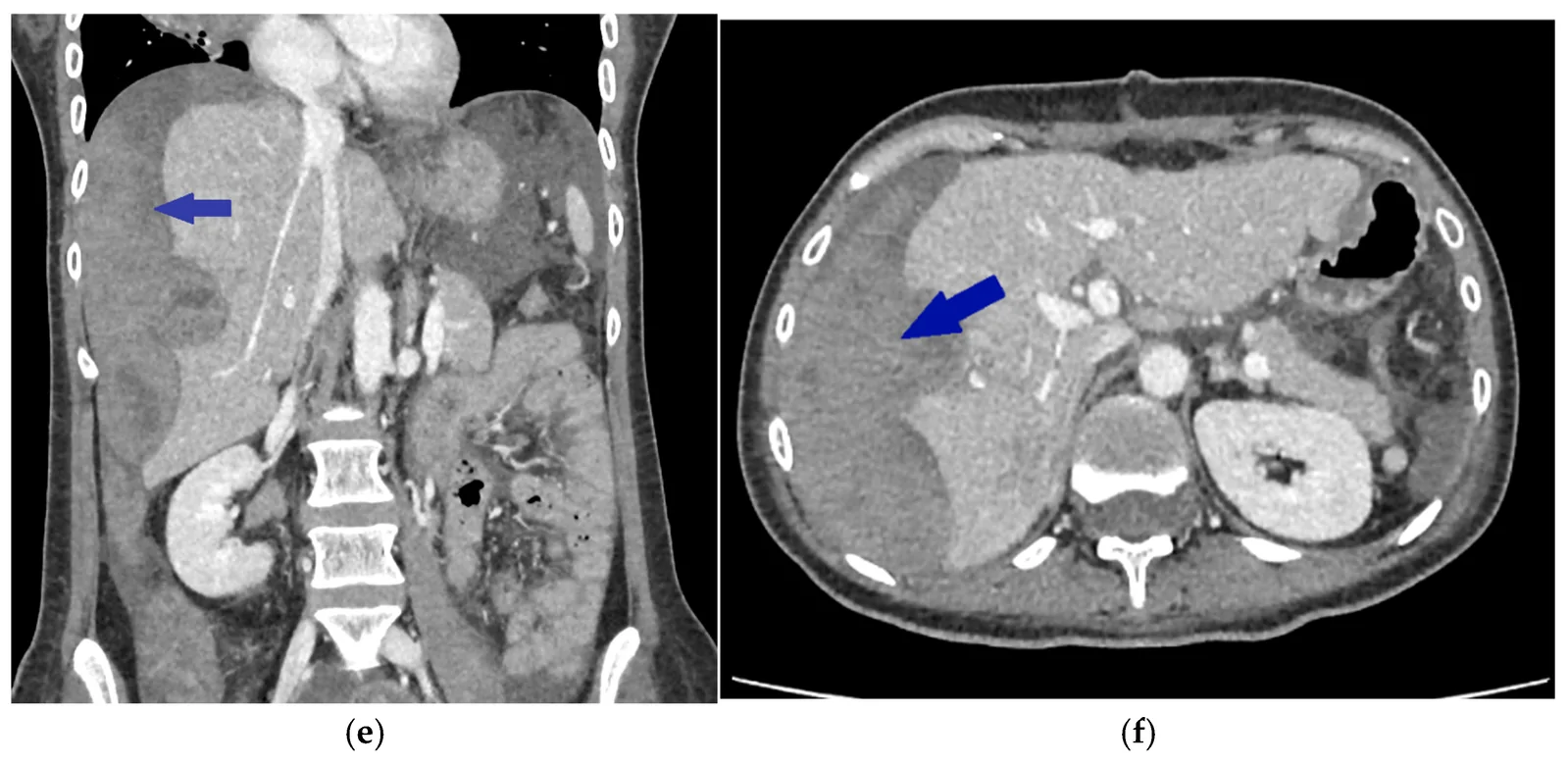
While recovering in the ICU, the patient reported the possibility of pregnancy. A gynaecological examination and transvaginal ultrasonography excluded the possibility of pregnancy despite an increase in βhCG levels (29,122 mIU/mL; after 48 h, 36,201 mIU/mL; normal < 5 mIU/mL in non-pregnant women). No ovarian or pelvic lesions suggestive of a gonadal primary tumour were identified. A few days later, another haematoma was detected in the right lobe of the liver- marked with blue arrow (**e**,**f**), and the patient underwent a laparotomy. Histopathological examination of the liver tissue confirmed the presence of metastatic foci of choriocarcinoma (immunohistochemical findings: CGT (+), Pancytokeratin (+), Ki67 about 60–70% positive cells, Inhibin (+), β-catenin (-), HepPar (-).

**Figure 6:**
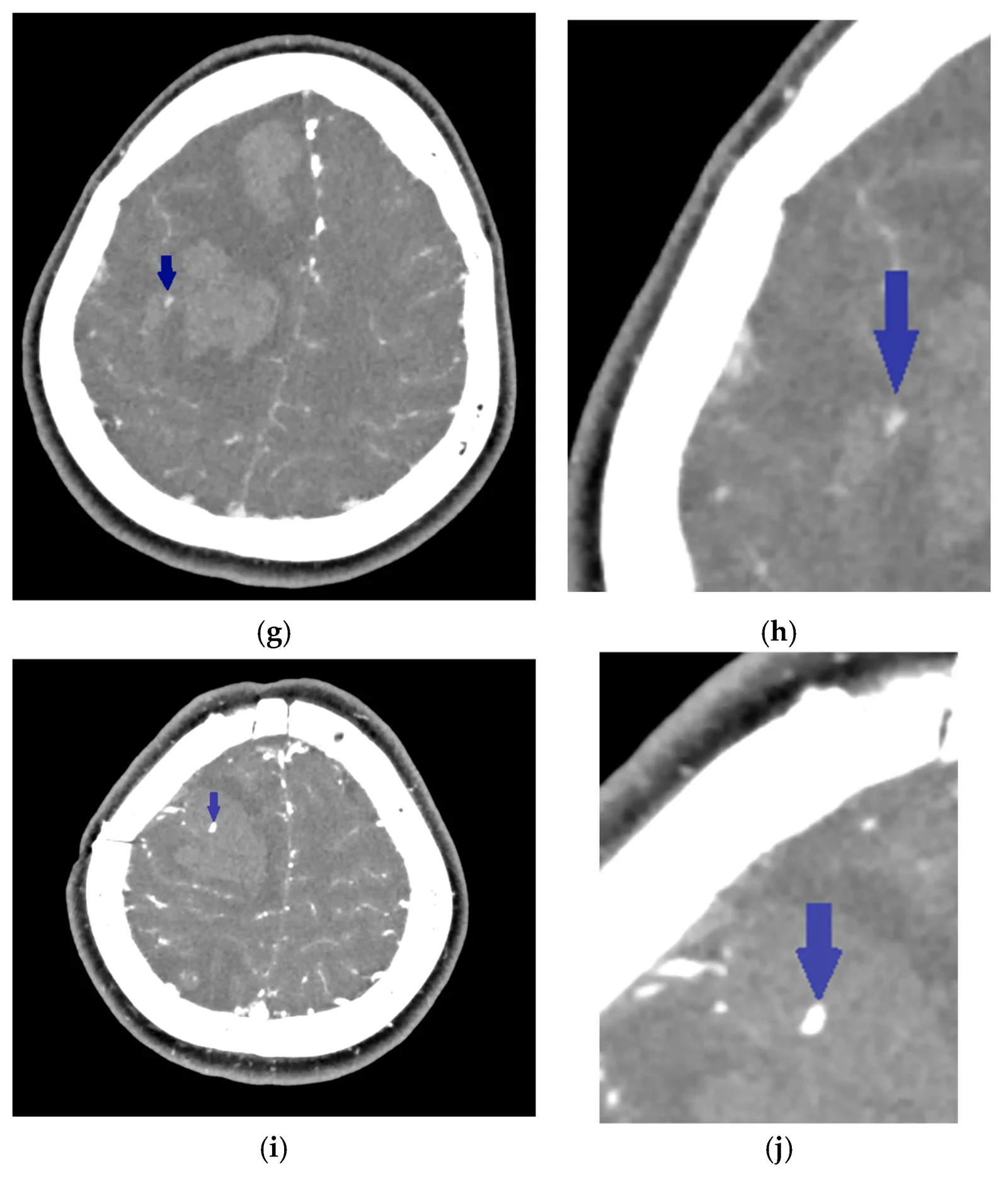
Control CT angiography of the head revealed two round contrast-enhancing vascular lesions—marked with blue arrow (**g**) first lesion, (**h**) zoomed first lesion; (**i**) second lesion, (**j**) zoomed second lesion) up to 2 mm. In the clinical context, they were interpreted as neoplastic aneurysms associated with choriocarcinoma. The patient with left-sided hemiparesis was then transferred to an oncologic hospital for further treatment. Based on the FIGO/WHO Prognostic Scoring System for Gestational Trophoblastic Neoplasia, the patient had a score of 19, classifying her as ultra-high risk [1]. An ultra-high risk score and brain involvement had directly influenced treatment decisions. Standard first-line chemotherapy EMA-CO was not initiated. She received salvage chemotherapy (etoposide, cisplatin) induction before starting the main EP-EMA regimen (etoposide, cisplatin, methotrexate, dactinomycin). During chemotherapy, no surgical or radiotherapeutic procedures were performed. There was a decrease in the serum β-hCG concentration to 0.7 mIU/mL by the end of chemotherapy. Throughout treatment, a head CT showed less brain oedema and regression of haemorrhagic changes. Additionally, the mass in the right lung decreased from 58 × 70 mm to 40 × 35 mm. The control CT scan of the abdomen demonstrated no signs of pathological enhancement, metastatic malformations or new bleeding. During the one-year follow-up, the patient had persistent left-sided paresis, was moving in a wheelchair and was undergoing neurological rehabilitation; no recurrence of bleeding was observed, and the mass in the lung did not increase. It is worth noting that the MRI scan was performed before the first episode of CNS haemorrhage During the one-year follow-up, the βhCG concentration was 0.2 mIU/mL. Choriocarcinoma is a highly malignant trophoblastic tumour characterised by the secretion of human chorionic gonadotropin (β hCG) [2]. Depending on its origin, choriocarcinoma is divided into gestational (GCC) or nongestational (NGCC). Although most cases are gestational, nongestational cases should not be overlooked in the differential diagnosis. Estimates indicate that 50% of gestational choriocarcinomas may develop after complete moles, as well as after normal pregnancies, miscarriages, partial hydatidiform moles or ectopic pregnancies [3]. In contrast, the origin of NGCC remains ambiguous. Choriocarcinoma is a rare and aggressive malignancy that may first become apparent through hemorrhagic metastatic disease. Although β-hCG is a key diagnostic marker, it is not routinely included in the initial assessment of patients with unexplained hemorrhagic lesions. Histologically, choriocarcinoma was confirmed, but due to the similarity of the microscopic appearance, its origin could not be determined [4]. The problem with confirming GCC or NCGO in the reported case was the lack of genotyping tumour DNA and comparison with the patient’s DNA. Detection of paternal DNA using STR analysis is crucial to gestational origin [5,6,7]. The patient’s molecular testing was not performed because it was unavailable. The patient had a history of delivery, intermenstrual bleeding before hospitalisation, and suspected pregnancy, which may suggest a gestational aetiology. The primary site of the choriocarcinoma could not be identified. In our case, failure to visualise a tumour mass in the uterus and ovary does not determine the diagnosis. A potential lesion could be regressed. Another option is a nongestational choriocarcinoma. The mass in the right lung, with unknown histopathology, could have been the primary lesion. Cases of a primary pulmonary choriocarcinoma (PPC) have been reported in the literature [8,9]. PPC is a rare and aggressive malignancy that may first become apparent through hemorrhagic metastatic complications, as in our case. Diagnosing choriocarcinoma is challenging. In some cases, the ^18^F-FDG PET/CT can be used, allowing for full recognition of atypical locations and rare trophoblastic tumours [10,11]. Choriocarcinoma must be included in the differential diagnosis when multiple sources of haemorrhage are identified. β-hCG is a highly available marker and should be routinely assessed.

## Data Availability

Original data will be available upon a justified request, from the corresponding author, after obtaining patient’s consent.
